# Spontaneous Uterine Perforation Caused by Pyometra: A Case Report

**DOI:** 10.5812/ircmj.14491

**Published:** 2014-08-17

**Authors:** Zohreh Yousefi, Noorieh Sharifi, Maryam Morshedy

**Affiliations:** 1 Ghaem Hospital, Faculty of Medicine, Mashhad University of Medical Sciences, Mashhad, IR Iran

**Keywords:** Pyometra, Uterine Perforation, Acute Abdomen, Postmenopause

## Abstract

**Introduction:**

Pyometra is an accumulation of purulent material or pus in the uterine cavity. Spontaneous perforation of uterus by pyometra is rare. This is a clinical presentation and management of a spontaneous perforation of uterine caused by pyometra.

**Case Presentation:**

This is a case report on spontaneously perforated associated with pyometra secondary to cervical malignancy. The patient underwent exploratory laparotomy with total hysterectomy and bilateral salpingo-oophorectomy.

**Conclusions:**

Spontaneous rupture of pyometra duo to cervical cancer in cases of acute abdomen in elderly patients should be considered.

## 1. Introduction

Pyometra or collection of purulent fluid in the uterine cavity is an uncommon condition (1). The literature review showed that the incidence of pyometra was 0.2% to 5% in all gynecologic admissions and occurred in 13.6% disorders of elderly gynecologic outpatients (2). So far, 30 cases of pyometra have been reported (3). Pyometra can be caused under a number of gynecological conditions either malignant or benign that lead to cervical stenosis. These possible factors include endometrial polyp, leiomyoma, cervical or endometrial carcinoma, and infection especially senile cervicitis (4, 5). However other factors can be considered as following conditions such as a forgotten IUD, cervical occlusion after surgery, and radiation (6). Furthermore, idiopathic reasons should be noted. In addition, pyometra occurs in old women with higher incidence of concurrent medical conditions (7). Moreover, these patients have a critical condition ,since there is the probability of catastrophic spontaneous perforation of uterus resulting in significant morbidity and mortality (8). This case report aimed to present a spontaneous rupture of pyometra and generalized peritonitis caused by cervical tumor.

## 2. Case Presentation

A 70-year-old woman Gravid 12 was referred to the Tumor Clinic of Ghaem Hospital, Mashhad University of Medical Sciences in September 2013. Her symptoms were abdominal pain, loss of appetite, nausea, and vomiting from 20 days ago with a history of moderate hematemesis. No severe disease was observed in her medical records. She looked dehydrated and ill in terms of general appearance. Vital signs were blood pressure 70/50 Hgmm, pulse rate 100/minute, temperature 36.1°C and respiratory rate 14/minute. Laboratory studies detected leukocytes 20000 and PMN 87%. Liver and kidney function tests were normal. She was misdiagnosed with the left bundle block view in ECG and the AF rhythm in auscultation which caused her to undergo medical therapy in CCU. At first admission in hospital, abdominal examination revealed tenderness in the right and left lower quadrant of abdomen without rebound tenderness and guarding, and evidence of rigidity and abdominal distention.

**Figure 1.**
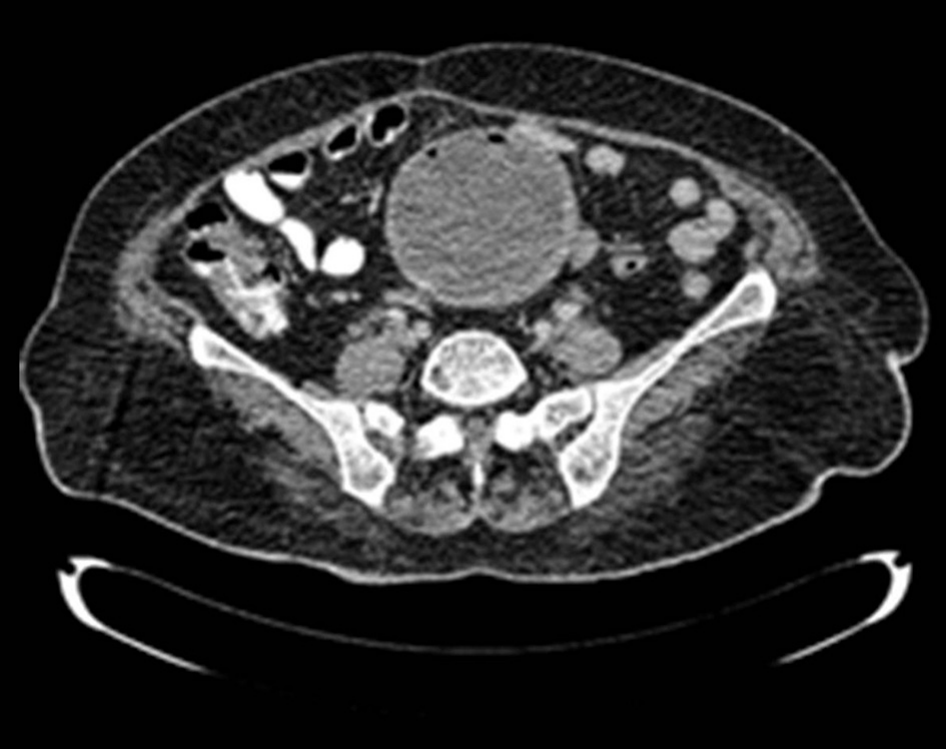
CT-Scan: A round mass, cystic lesion and focal calcification at its periphery, probably ovarian cyst adenoma.

**Figure 2.**
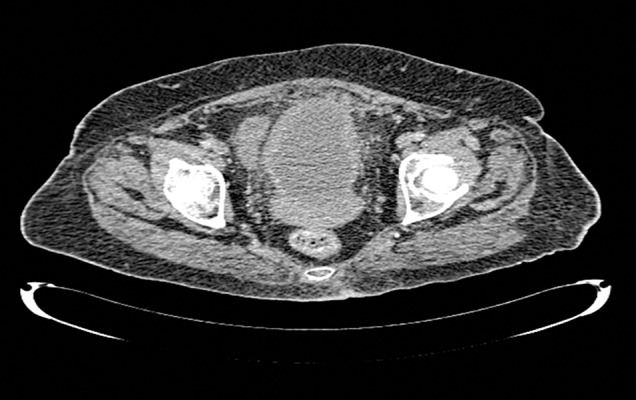
CT-Scan: Mass, a 14 × 11.5 cm cystic lesion in pelvic cavity without significant change in post-contrast images.

In sonography evaluation, size of uterus was 133.5 × 80.5 cm with hypoechoic mass 101 × 109 cm in the left lateral of uterine body, which could be probably interpreted as uterine myoma, and some free fluid in peritoneal cavity was also reported. Paracentesis 5 mL suppurative fluid was aspirated under the guidance of sonography. Findings of CT-scan were a round cystic mass with focal calcification at its periphery located in the pelvic cavity with the diameter about 14 × 11.5 cm probably ovarian cyst adenoma (Figures 1 and 2).

Based on the diagnosis of peritonitis (suppurative fluid aspirated), emergency laparotomy was performed and 700 mL of suppurative fluid in the abdominal cavity with normal appearance bowel and liver was found. The origin of pus draining was a 1 × 1 cm rupture in the anterior wall of uterus. However, there were multiple polypoid tumors in cervical canal yielded stenosis of cervical discharge. After peritoneal irrigation with 5 to 6 L of normal saline, total hysterectomy with bilateral salpango-oophorectomy was performed. Then, the patient received board-spectrum antibiotic. But in recovery image, the patient became oliguria with rise of creatinine level, and decrease of the blood pressure, despite normal hemoglobin level. Despite all medication and efforts performed, the general condition of the patient deteriorated and she expired ten hours after operation probably because of the septic or cardiogenic shock.

## 3. Discussion

Pyometra is also an unusual cause of peritonitis in postmenopausal women. Spontaneous perforated pyometra is a rare entity. In cervical cancer, generalized peritonitis due to pyometra is extremely rare and only four cases have been reported (9). A possible diagnosis in the elderly women with an acute abdomen, especially with underling of genital malignancy, should be considered (10). In most cases, spontaneously perforated pyometra has been diagnosed intra-operatively. The patient under study was diagnosed with features of acute abdomen and generalized peritonitis. Comparison of the findings concerning spontaneous uterine perforation cases are summarized in (Table 1) (11-13).

**Table 1.** Characteristics of the Patients With Spontaneous Uterine Perforation Caused by Pyometra ^a^

Author	Age, y	Clinically Diagnosis	Treatment	Histopathologic Examination	Malignancy
**Saha PK**	60	pG I T	sub TAH with BSO + PP	gangrene and infection	no
**Geranpayeh L**	63	pG I T	TAH with BSO + PP	uterine infection	no
**Inui A1**	88	peritonitis	sub TAH with BSO + PP	pyometra	no
**Sahoo SP**	50	hollow viscus perforation	TAH with BSO + PP	uterine infection	no
**Present case**	70	peritonitis	TAH with BSO + PP	Pyometra	yes

^a^ Abbreviations: TAH, total abdominal hysterectomy; BSO, bilateral salpingo-oophorectomy; PP, peritoneal lavage; pG I T, Perforation of the gastrointestinal tract

**Figure 3.**
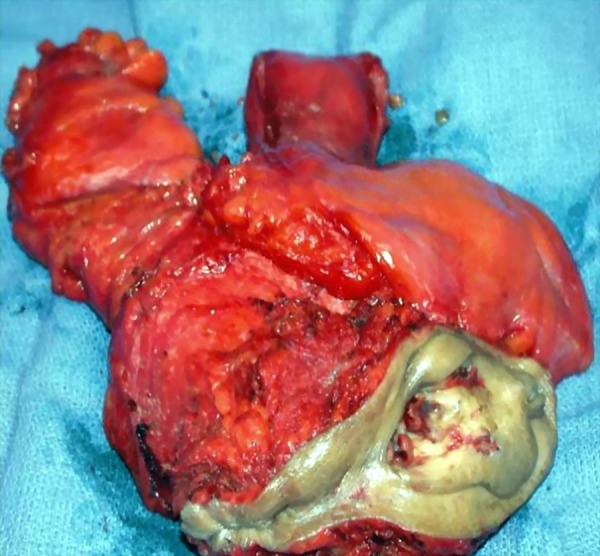
Macroscopic view of Ruptured Uterus Associated with Cervical Carcinoma and Pyometra

**Figure 4.**
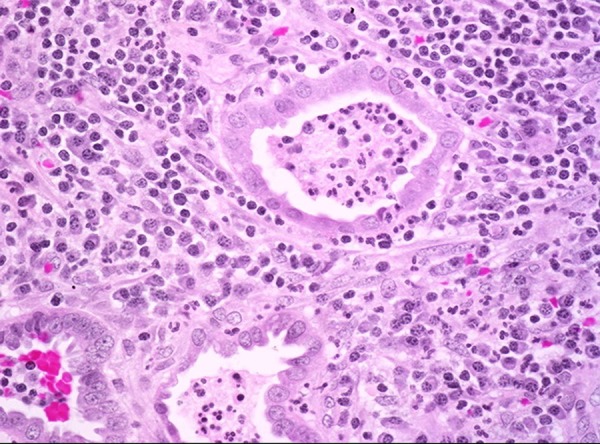
Uterine Wall With Extensive Necrosis and Fibrinoleukocyter Exudates (H × E 100 ×)

Stenosis of cervical canal and degenerative or necrotic processes in the uterine wall led to spontaneous perforation of pyometra (4). The classic symptoms of these patients are triad purulent vaginal discharge, lower abdominal pain, and postmenopausal bleeding. Indeed, nonspecific symptoms are common including vomiting, fever and uterine enlargement. It appears that more than 50% of all patients with non-ruptured pyometra are asymptomatic (14). The etiological organisms reported in bacterial cultures from the peritoneal cavity of pyometra were *Escherichia coli* and anaerobes such as Bacteroides, Peptococcus and Streptococcus species (15). The initial modality after diagnosis of uterine perforation is abdominal sonography , also overlapping of bowel loops in myometer or endometrial cavity may be missed with the findings of perforation (12). Criteria of ultrasonography in the current case were hypoechoic mass, probably uterine myoma and some free fluid in peritonea cavity. Additional diagnostic radiographic evaluations used for acute abdomen are CT-Scan and MRI. But, these instruments are not recommended, except in morbid conditions (16). Unfortunately, CT-Scan was not useful for this patient. Generally, the final diagnosis is obtained by exploratory laparotomy, and perforation of uterus diagnosed intra-operatively (17). Pathological investigation of the surgical specimen of our patient revealed ruptured myometrial wall with pyometra and invasive cervical carcinoma (Figures 3 and 4)

Treatment of ruptured pyometra in patients with cervical cancer depends on the clinical condition of the patient and the preoperative diagnosis (18). The best approach for ruptured pyometra is emergency laparotomy, irrigation of peritoneal cavity, and then simple hysterectomy. However, in unruptured cases of pyometra, cervical dilatation and drainage must be considered (2). In cases of preserve fertility, irrigation of abdominal cavity after evacuation of the uterine cavity and the repair of uterine perforation should be considered (19). Our patient was treated by removing ovaries, hysterectomy and irrigation of abdominal cavity. Based on the above explanations, to prevent recurrent disease regular monitoring should be performed after initial treatment.

It must be noticed that spontaneous uterine perforation associated with pyometra due to underling malignancy is a serious medical condition. The majority of these patients are old and most of them are associated with poor general condition that yields to significant morbidity and mortality. Therefore, it is recommended to manage these patients without delay.
